# The Implementation of New Technologies Deserves Our Particular Attention Towards Radiation Safety

**DOI:** 10.5334/jbr-btr.1078

**Published:** 2016-03-31

**Authors:** Nico Buls

**Affiliations:** 1 UZ Brussel - Vrije Universiteit Brussel, BE

**Keywords:** radiation, safety, protection, exposure, dose, interventional

The use of radiation in medicine has led to major improvements in the diagnosis and treatment of human diseases. Annually, worldwide, more than 3,600 million X-ray examinations are performed, 37 million nuclear medicine procedures are carried out, and 7.5 million radiotherapy treatments are given [1]. As the benefits for patients gain recognition, the use of radiation in medicine increases. Radiologists and other doctors that use X-rays have a special mission to avoid unjustified or nonoptimized use of radiation since they are responsible for the largest source of man-made ionizing radiation to the population and to exposed healthcare workers. Recent data from 22 European countries shows that over 68 percent of all radiation-monitored workers are within the medical field [2]. This is a very large portion compared to the second and third runners-up, namely, the nuclear field (11%) and the industry (8%).

Considering these numbers, it is not surprising that the medical field is also associated with a relatively high collective radiation dose for its workers. This collective dose unit simply equals the sum of all individual worker doses and can consequently be used to describe the extent to which a group of people has been exposed. In no other professional field are workers more exposed to ionizing radiation than ours. According to the same European data source [2], 21 percent of the annual collective radiation dose goes to healthcare workers. Professionals in the industry and nuclear field receive 12 percent and 17 percent, respectively. Although we can argue that these few numbers merely provide a simplistic approach – in reality, occupational radiation doses are very heterogeneously distributed, with some specialties receiving relatively high doses and others receiving almost nothing – they do underline the importance of radiation protection for workers in medicine. Particularly, the interventional radiologist is frequently exposed to elevated levels of scatter radiation. In high-volume cath labs, the most active and experienced doctors have an annual effective dose of around 5 mSv, and a professional lifetime attributable excess cancer risk of 1 in 100 [3]. Interventional radiologists receive similar doses [4].

There are two main biological effects of radiation: stochastic effects, which include carcinogenic effects, and direct tissue reactions, which can only occur when the dose exceeds a certain threshold. The most reported example of radiation-induced tissue reactions among doctors is cataract. Recently, several epidemiological studies among surveys of staff in interventional rooms report an increased incidence of lens opacities and even suggest a nonthreshold effect [5], indicating that the eye lens is more radiosensitive than previously considered. In view of these results, the International Commission of Radiation Protection (ICRP) immediately recommended in their Publication 118 to reduce the eye lens dose limit from 150 mSv to 20 mSv in a year. A drastic reduction which is, until today, unfortunately not yet implemented in our Belgian legislation.

In the recent past, healthcare professionals had a low awareness of radiation doses in radiological medical procedures as well as of the nature and magnitude of the related radiation risks. An area of particular concern remains the implementation of new techniques. While the development of modern health technology makes these new applications safer, their inappropriate use can lead to unnecessary or unintended radiation doses and can cause potential health hazards for patients and staff.

In the *JBSR* this month, Braak et al. report on the radiation safety of such a new technique [6]. They investigate scatter exposure to the interventional radiologist during procedures with cone-beam CT guidance (CBCT), a novel technique that became available with the introduction of flat-panel detectors in the angiosuite. Compared to a standard 2D fluoroscopic acquisition, a rotational CBCT geometry will provoke a more complex scatter field to the radiologist, potentially resulting in elevated radiation doses. The authors characterize this exposure to the interventionalist in a prospective study and demonstrate that doses can be drastically reduced by taking simple measures, such as placing a protective lead drape on the patient or by using ceiling-mounted shielding. With their study, the authors help to put this new technique into practice, and they have set another example for promoting a safe and effective use of radiation in medicine.

## Competing Interests

The author declares that they have no competing interests.
